# Spatial patterns of immunogenetic and neutral variation underscore the conservation value of small, isolated American badger populations

**DOI:** 10.1111/eva.12410

**Published:** 2016-08-21

**Authors:** Yessica Rico, Danielle M. Ethier, Christina M. Davy, Josh Sayers, Richard D. Weir, Bradley J. Swanson, Joseph J. Nocera, Christopher J. Kyle

**Affiliations:** ^1^ Forensic Science DepartmentTrent UniversityPeterboroughONCanada; ^2^ Natural Resources DNA Profiling and Forensics CentreTrent UniversityPeterboroughONCanada; ^3^ Ontario Badger ProjectGuelphONCanada; ^4^ Department of Integrative BiologyUniversity of GuelphGuelphONCanada; ^5^ Ecosystems Protection & Sustainability BranchMinistry of EnvironmentVictoriaBCCanada; ^6^ Central Michigan UniversityMount PleasantMIUSA; ^7^ Wildlife Research and Monitoring SectionMinistry of Natural Resources & ForestryPeterboroughONCanada; ^8^ Present address: CONACYTInstituto de Ecología A.C.Centro Regional del BajíoAvenida Lázaro Cárdenas 253PátzcuaroMichoacán61600México

**Keywords:** conservation genetics, gene flow, local adaptation, Mustelids, northern range, selection, small isolated populations

## Abstract

Small and isolated populations often exhibit low genetic diversity due to drift and inbreeding, but may simultaneously harbour adaptive variation. We investigate spatial distributions of immunogenetic variation in American badger subspecies (*Taxidea taxus*), as a proxy for evaluating their evolutionary potential across the northern extent of the species’ range. We compared genetic structure of 20 microsatellites and the major histocompatibility complex (MHC DRB exon 2) to evaluate whether small, isolated populations show low adaptive polymorphism relative to large and well‐connected populations. Our results suggest that gene flow plays a prominent role in shaping MHC polymorphism across large spatial scales, while the interplay between gene flow and selection was stronger towards the northern peripheries. The similarity of MHC alleles within subspecies relative to their neutral genetic differentiation suggests that adaptive divergence among subspecies can be maintained despite ongoing gene flow along subspecies boundaries. Neutral genetic diversity was low in small relative to large populations, but MHC diversity within individuals was high in small populations. Despite reduced neutral genetic variation, small and isolated populations harbour functional variation that likely contribute to the species evolutionary potential at the northern range. Our findings suggest that conservation approaches should focus on managing adaptive variation across the species range rather than protecting subspecies per se.

## 1 Introduction

Habitat loss and climate change are major threats to global biodiversity, rapidly altering environmental selective pressures to which species must adapt to persist in their local environments. Genetic diversity is a necessary attribute for local adaptation and is influenced by the forces of gene flow, genetic drift and selection, which vary across a species’ range (Garant, Forde, & Hendry, 2007; Kawecki 2008). Populations that are small and geographically isolated often have low genetic diversity and high genetic differentiation compared to large and well‐connected populations, because of their small effective population sizes (*N*_e_), reduced gene flow and higher rates of genetic drift and inbreeding (Eckert, Samis, & Lougheed, 2008; Munwes et al., 2010; Wagner et al., 2012). These characteristics suggest that small and isolated populations are more likely to go extinct before they can adapt to new environmental conditions (Bijlsma & Loeschcke, 2012). Because economic and logistic resources in conservation management are limited, it has been argued that prioritization of populations for conservation should target less vulnerable populations such as those with large abundances and high genetic diversity (Jamieson & Allendorf, 2012; Lesica & Allendorf, 1995). On the other hand, local selective pressures and restricted gene flow can also drive local adaptation in small and isolated populations (Lenormand, 2002; Vucetich & Waite, 2003), which implies that these populations are also relevant for conservation because of their unique adaptive genetic potential (Mayr, 1954; Petren, Grant, Grant, & Keller, 2005). There is an increasing recognition that conservation management should, as much as possible, focus on maintaining the adaptive genetic diversity within a species’ range by understanding the processes shaping their evolutionary potential (Eizaguirre & Baltazar‐Soares, 2014).

Hence, effectively mitigating a species’ declines due to anthropogenic‐related threats requires an understanding of both spatial distributions of genetic variation and patterns of local adaptation to predict how populations may respond to changing selective pressures (Bourne et al., 2014; Gibson, Van der Marel, & Starzomski, 2009). The success of interventions such as translocations, assisted migration and genetic rescue depends strongly on maximizing individual fitness in a novel environment. Ideally, genetic data should inform such interventions (Hedrick, 2014). Neutral genetic markers, such as microsatellites, provide information about gene flow and demography (Kirk & Freeland, 2011), but do not accurately predict adaptive genetic potential (Reed & Frankham, 2003; Volis, Ormanbekova, Yermekbayev, Song, & Shulgina, 2015). Thus, adaptive genetic variation should be assessed through examinations of highly polymorphic, functional markers that respond directly to selective pressures (Holderegger, Buehler, & Gugerli, 2010; Kirk & Freeland, 2011).

The major histocompatibility complex (MHC) is the most polymorphic gene family in vertebrates and has a key role in the adaptive immune response against a wide range of pathogens (Piertney & Oliver, 2006). Given these attributes, MHC loci have been used as an ideal candidate for investigating adaptive immunogenetic variation in wildlife populations (Wegner, Reusch, & Kalbe, 2003; Kyle et al., 2014; Savage and Zamudio 2016). Evidence suggests that MHC genes can evolve via sexual selection, influencing offspring fitness (Jan Ejsmond, Radwan, & Wilson, 2014; Sin et al., 2015). Moreover, MHC genes can respond to selective pressures mediated by pathogens via heterozygote advantage, negative frequency‐dependent selection and heterogeneous selection promoting local adaptation (Bernatchez & Landry, 2003; Piertney & Oliver, 2006; Spurgin & Richardson, 2010). These mechanisms are not mutually exclusive and can shift over temporal and spatial scales, or act synergistically (Dionne, Miller, Dodson, & Bernatchez, 2009; Herdegen, Babik, & Radwan, 2014; Oliver, Lambin, Cornulier, & Piertney, 2009). Erosion of MHC diversity might represent a serious risk to vulnerable populations as it can increase disease susceptibility (Garrigan & Hedrick, 2003; Goyette et al., 2015).

The relative effects of selection, gene flow and genetic drift on MHC can be disentangled by contrasting genetic structure at MHC and neutral genetic loci (Ekblom et al., 2007; Kyle et al., 2014; Spurgin & Richardson, 2010). For instance, balancing selection through heterozygote advantage or negative frequency‐dependent selection maintains MHC polymorphism and counteracts the loss of rare alleles by genetic drift (Kamath & Getz, 2012; Rico, Morris‐Pocock, Zigouris, Nocera, & Kyle, 2015; Strand et al., 2012). This scenario predicts that genetic differentiation among populations is weaker for MHC genes than for neutral loci (Tobler et al., 2014; Van Oosterhout et al., 2006). Conversely, signatures of adaptive divergence at MHC relative to neutral loci (Dionne et al., 2009; Herdegen et al., 2014; Oliver et al., 2009) indicate heterogeneous selection caused by variation in local selective pressures. On the other hand, genetic drift has been described as the main evolutionary force shaping MHC polymorphism in small, isolated populations (Luo, Pan, Liu, & Li, 2012; Munguia‐Vega et al., 2007).

**Figure 1:**
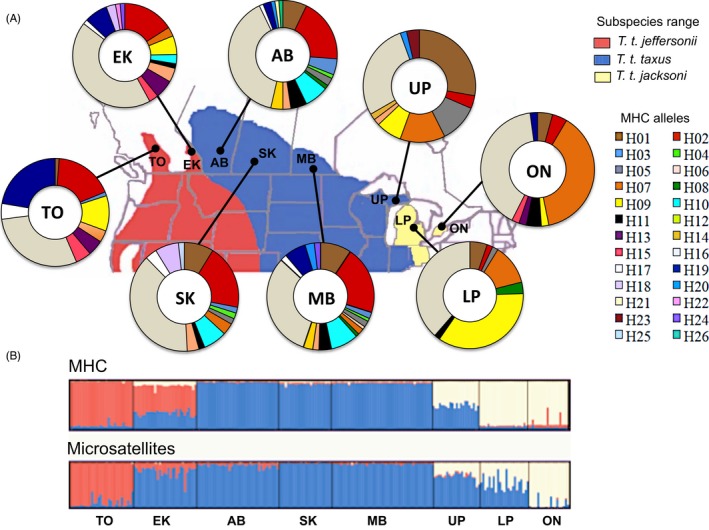
Distributions of genetic variations for MHC and neutral microsatellite loci across eight populations of American badger at the northern portion of its range. (A) Relative frequency distribution of 26 MHC alleles per population. Each colour of the pie chart represents an MHC allele, while its size is proportional to the frequency of that allele within a sampling location. (B) Bar plot of population membership scores for *k = 3* genetic clusters inferred with STRUCTURE based on 26 MHC alleles (top) and 20 neutral microsatellites (below). The different colours in the background map denote the subspecies ranges (see legend) from which populations were sampled: Thompson‐Okanagan (TO), East‐Kootenay (EK), Alberta (AB), Saskatchewan (SK), Manitoba (MB), Upper Peninsula (UP) and Lower Peninsula (LP) of Michigan, and Ontario (ON).

Here, we assess the spatial variation of MHC polymorphism relative to the spatial distribution of neutral genetic diversity to evaluate the evolutionary potential of small and geographically isolated populations and large and well‐connected populations of American badgers (*Taxidea taxus*), using populations across the northern extent of the species’ range. American badgers are divided into four subspecies based on differences in skull size, pelage colour and geographical distribution (Long, 1972). Evidence from microsatellites and mitochondrial DNA (mtDNA) showed that genetic substructure exists within subspecies (Ethier, Laflèche, Swanson, Nocera, & Kyle, 2012; Kyle, Weir, Newhouse, Davis, & Strobeck, 2004). Three recognized subspecies of *T. taxus* reach their northern range limits in Canada (Fig. 1). In British Colombia (BC), *T. t. jeffersonii* occurs in two small endangered, disjunct populations (Thompson‐Okanagan [TO] and East‐Kootenay [EK]), which are separated by mountain ranges (COSEWIC, 2012). In central Canada, larger populations of *T. t. taxus* occur from the prairies of Alberta (AB), Saskatchewan (SK) and Manitoba (MB). These populations are connected to *T. t. taxus* in the Great Plains of the United States and the Upper Peninsula (UP) of Michigan. Finally, a small, endangered, isolated population of *T. t. jacksoni* persists in southern Ontario (ON; Fig. 1 background map). This ON population is most closely related to *T. t. jacksoni* in the Lower Peninsula (LP) of Michigan. The most peripheral western and eastern populations (TO and ON) are also the most genetically differentiated (Ethier et al., 2012; Kyle et al., 2004).

In this study, we aim to investigate the conservation genetic value of peripheral populations of an endangered carnivore by evaluating the relative influences of selection and neutral processes as drivers of MHC polymorphism among and within badger subspecies at the northern extent of the species’ range. We compare spatial patterns of functional immunogenetic (MHC DRB exon 2) and neutral genetic (20 microsatellites) variations to account for the effects of gene flow and genetic drift. Specifically, we sought to determine whether small, isolated badger populations show lower levels of MHC diversity relative to large and well‐connected nonperipheral populations, resulting from high rates of genetic drift or whether these isolated peripheral populations act as repositories of local immunogenetic adaptation. We also sought to determine whether functional immunogenetic markers reflect current subspecies and conservation designations for *T. taxus*. Overall, these empirical data on the diversity of functional loci aid in evaluating the evolutionary potential of small, isolated populations and have the potential to better inform conservation strategies focusing on the maintenance and management of adaptive genetic variation.

## 2 Methods

### 2.1 Sample collection and microsatellite genotyping

American badgers are elusive and nocturnal animals that are difficult to observe in the wild, and hence, most of the samples used in this study were opportunistically collected (*n *=* *236) from hair snares, harvested pelts, incidental deaths and radio‐tagged badgers. Samples in Canada and the north‐western USA included *T. t. jeffersonii* from BC (TO, *n *=* *30; EK, *n *=* *30); *T. t. taxus* from AB (*n *=* *39), SK (*n *=* *25), MB (*n *=* *48) and UP (*n *=* *22); *T. t. jacksoni* in LP (*n *=* *23) and ON (*n *=* *19) (Fig. 1). Our samples included 193 samples that were collected in Kyle et al. (2004) and Ethier et al. (2012).

We genotyped samples at 20 loci (Primer pairs: Tt13, Tt15, Tt17, Tt20, Tt21, Tt22, Tt23, Tt27, Rico et al., 2014; Tt‐1, Tt‐2, Tt‐3, Tt‐4, Ma‐1, Ma‐15, Davis & Strobeck, 1998; Gg234, Duffy, Landa, O'Connell, Stratton, & Wright, 1998; Gg443, Gg465, Walker, Vilà, Landa, Lindén, & Ellegren, 2001; Mvis072, Fleming, Cook, & Ostrander, 1999; Mvis87, O'Connell, Wright, & Farid, 1996; MP0085, Jordan et al., 2007). We pooled amplification of microsatellites in five PCR multiplexes with fluorescent dyes (Table S1) following Rico et al. (2014). PCR products were run on an ABI 3730 Automated Sequencer (Applied Biosystems, Foster City, CA) with a 500 LIZ size standard. Electropherograms were analysed using GENMARKER 1.91 (SOFTGENETICS, State College, PA, USA). Genotyping error was assessed by re‐amplifying all sets of primers from independent DNA extractions of the same tissue sample in approximately 8% of total samples.

### 2.2 Major histocompatibility complex DRB‐2 amplification and genotyping

We used 454 sequencing to characterize MHC class II DRB, following Oomen, Gillett, and Kyle (2013). Amplicons of 185‐bp fragment were amplified using a modified reverse primer DRB‐3c (CCGCTGCACAGTGAAACTCTC, Murray & White, 1998) with a MID adaptor (MID1‐MID6, MID11; Roche Diagnostics) and modified forward DRB‐5c primer (TCAATGGGACGGAGCGGGTGC) with a MID adaptor (MID1‐MID8, MID10‐MID11, MID13‐MID16; Roche Diagnostics). Various combinations of the 14 MID's tags for individual identification provided 96 unique tags that differed by at least 6–10 bp. Pooled, MIT tagged libraries from 70 to 80 individuals were prepared for 454‐sequencing using a Roche GS Junior System. We sequenced 27 individuals on independent 454 sequencing runs to estimate MHC genotyping error.

We used jMHC (Stuglik, Radwan, & Babik, 2011) to extract and assign raw FASTA‐format reads to each individual. Sequences without complete primers and tags, sequences containing indels or ambiguous base pairs or sequences that did not match the expected allele size of 185 bp were discarded. Analysis of 454 sequencing data can be challenging because true MHC alleles must be distinguished from artefact sequences generated during PCR or 454 sequencing. To filter true alleles from artefacts, we applied two approaches: (i) the allele validation threshold following the multistep criteria described by Galan, Guivier, Caraux, Charbonnel, and Cosson (2010) and Sepil, Moghadam, Huchard, and Sheldon (2012) and (ii) the degree of change sequencing modelling by Lighten, Van Oosterhout, Paterson, Mcmullan, and Bentzen (2014). Both approaches rely on the assumption that artefacts are less frequent than true alleles (Lighten et al., 2014; Sepil et al., 2012). To apply the allele validation threshold method, we calculated the maximum per amplicon frequency (MPAF) for each variant, which is the maximum proportion of the individual's reads for a given variant among all individuals in which the variant was present. We ranked variants based on the highest MPAF values. We started filtering variants with the lowest MPAF (≤1%) to check whether they could be explained as artefacts based on point mutations (≤2 bp substitutions) from a sequence of higher frequency (MPAF >10%) within the same amplicon. This initial assessment indicated that sequences with MPAF ≤3% were artefacts. In contrast, variants with MPAF ≥10% were present in more than one individual from an independent run and were considered true alleles. Variants between MPAF ≥3%–10% were checked manually to determine whether they could be explained by a difference of 1–2 bp from a parental true allele, contained premature stop codons, or produced a frame‐shift mutation. Using the degree of change sequencing modelling approach, variants were ranked based on the number of reads per amplicon to calculate the cumulative sequencing depth among ranked variants. Variants with the highest degree of change were used as a basis to calculate statistical breakpoints between artefacts and true alleles based on sequencing depth models for each amplicon. We used the Excel Macro for MHC genotyping by Lighten et al. (2014). We contrasted the genotyping reliability of both models using the duplicated samples.

### 2.3 Data analysis: major histocompatibility complex

#### 2.3.1 Test for selection and recombination

We tested for signatures of historical positive selection in MHC using the one‐tailed *Z*‐test and likelihood codon‐based approaches. In the one‐tailed *Z*‐test, the selection parameter ω quantifies the ratio of nonsynonymous (*d*_*N*_) to synonymous (*d*_*S*_) substitutions, where ω *= d*_*N*_ */d*_*S*_ >1 indicates the effect of positive selection. We calculated the ratio of *d*_*N*_ and *d*_*S*_ per site, using the modified Nei–Gojobori method with the Jukes–Cantor correction for multiple substitutions in MEGA v6.6 (Tamura, Stecher, Peterson, Filipski, & Kumar, 2013). We calculated each of the above statistics for all codons, peptide‐binding regions (PBR) sites and non‐PBR sites separately, because PBR associated with pathogen binding are expected to be under strong selection. We identified putative PBR sites based on human MHC II molecular structure (Brown et al., 1993; Stern et al., 1994). Codon‐based maximum‐likelihood methods of balancing selection were implemented in Codeml in PAML (Yang, 2007). We tested six models allowing for different selection intensities among sites: M0 (one ratio ω), M1a (nearly neutral), M2a (positive selection), M3 (discrete), M7 (nearly neutral with beta distribution approximating ω variation) and M8 (positive selection with beta distribution approximating ω variation). We used likelihood ratio tests (LRT) to compare three nested models: M0 versus M3, M1a versus M2a, M7 versus M8; and determine the best fit to our data for presence of positive selection in models M3, M2a and M8. Positively selected sites were identified by Bayes empirical Bayes procedure (BEB) for models M2a and M8. Additionally, we conducted the fixed effects likelihood (FEL), random effects likelihood (REL) and the mixed effects model of evolution (MEME) tests implemented in the HyPhy software (hosted at Datamonkey: http://www.datamonkey.org/). We checked for signatures of recombination using the genetic algorithm recombination detection method using the Datamonkey website.

#### 2.3.2 Major histocompatibility complex diversity

Copy number variation of MHC loci within individuals occurs in numerous species (e.g. Sepil, Lachish, Hinks, & Sheldon, 2013; Zagalska‐Neubauer et al., 2010), complicating the assignment of amplified alleles to specific loci (Lighten et al., 2014). It became clear during our analysis that one to four alleles occurred within individual badgers, indicating the presence of at least two MHC DRB exon 2‐like loci. Therefore, we could not assign alleles to specific loci, requiring different measures of MHC diversity. At the population level, we estimated the mean number of pairwise differences (*k*) for each population in ARLEQUIN v3.11 (Excoffier, Laval, & Schneider, 2005). We also calculated the relative frequency of MHC alleles by counting the number of individuals carrying a particular allele divided by the total number of alleles in each population (Ekblom et al., 2007). At the individual level, we calculated MHC individual diversity as the number of alleles per individual divided by the maximum number of alleles found within individuals in the total data set (*n *=* *4). We tested for significant differences in MHC individual diversity among populations using a type II ANOVA followed by pairwise comparisons (Tukey's HSD, family‐wise α = .05). We tested the correlation between microsatellite allelic richness and MHC individual diversity using Pearson product moment correlations in R (R Core Team, 2014).

### 2.4 Data analysis: microsatellites

Significant departures from Hardy–Weinberg equilibrium (HWE) and linkage disequilibrium (LD) for each sampling location were examined using probability test in GENEPOP v.4.2 (Rousset, 2008). For each location, we estimated observed (*H*_o_) and expected heterozygosity (*H*_e_) using GENALEX v.6.5 (Peakall & Smouse, 2012), inbreeding coefficient (*F*_IS_) in FSTAT v.2.9.3.2 (Goudet, 1995) and allelic (*A*_r_) and private allelic richness (*P*_ar_) adjusted for unequal sample sizes by rarefaction in HP‐RARE v.1.0 (Kalinowski, 2005). We tested for significant differences in neutral genetic diversity (*A*_r_ *, H*_o_) between small, peripheral populations (TO and ON) and nonperipheral populations using 1,000 permutations in FSTAT v.2.9.3.2 (Goudet, 1995). We estimated *N*_e_ using the software LDNe (Do et al., 2014) by selecting the linkage disequilibrium and molecular co‐ancestry methods. Confidence intervals were estimated by jackknife resampling with 1,000 iterations.

### 2.5 Comparisons between neutral and functional markers

Pairwise MHC *F*_ST_ distances between all sampling regions pairs were calculated using the Jukes–Cantor distance model in ARLEQUIN by entering the nucleotide MHC sequences and the number of individuals per sequence and population as haplotype data. For microsatellites, *F*_ST_ was calculated using the number of different alleles. Statistical significance (*p *<* *.05) of *F*_ST_ values was estimated by 1,000 randomizations. We used partial Mantel correlations to test the effect of isolation by geographical distance (IBD) on MHC genetic distances, while controlling for the genetic differentiation at neutral loci. Significance of Mantel correlation coefficients was tested by permuting observations 1,000 times using the R library vegan (Oksanen et al., 2013).

To facilitate comparison between the two types of markers, we treated MHC and microsatellite data as dominant markers with alleles coded in a binary form (presence 1/absent 0) (Herdegen et al., 2014; Kyle et al., 2014; Nadachowska‐Brzyska, Zieliński, Radwan, & Babik, 2012). Population genetic structure based on the binary‐encoded data was analysed with Bayesian clustering analyses in STRUCTURE v2.3 (Pritchard, Stephens, & Donnelly, 2000) allowing for genetic admixture and correlated allele frequencies for 200,000 burn‐in steps followed by 400,000 postburn MCMC iterations. We tested scenarios ranging from 1 to 8 clusters (*k*), with 10 iterations at each value of *k*. We compared models with and without the LOCPRIOR function, which includes the sampling regions of origin in the analysis. The most likely number of *k*‐clusters was chosen by compiling runs using STRUCTURE HARVESTER v.0692 (Earl & vonHoldt, 2012), and assessing the increase in *P*_r_ (*X*|*K*) (Pritchard et al., 2000) and using the ad hoc ∆*K* method (Evanno, Regnaut, & Goudet, 2005). Individual membership probabilities of the inferred *k*‐clusters from 10 independent replicates were averaged using CLUMPP v.1.1.2 (Jakobsson & Rosenberg, 2007), and clusters were visualized using DISTRUCT v.1.1 (Rosenberg, 2004). We performed analyses of molecular variance (AMOVA), by partitioning the genetic variance among the eight sampling regions and the three subspecies groups. Significance of AMOVA components was tested with 1,000 permutations in GENALEX (Peakall & Smouse, 2012). To evaluate the influence of neutral processes on population genetic differentiation at MHC, we performed a co‐inertia analysis (CoA) to assess the joint structure of MHC and microsatellite loci. CoA is a multivariate method that assesses the covariance structure between data sets having the same observations (Dray & Dufour, 2007). This multivariate method is not limited to population‐pair comparisons such as *F*_ST_ and does not rely on mutational, HWE and LD equilibrium assumptions (Jombart, 2008). For each binary‐matrix, we performed a factorial PCA using sampling regions as predefined groups, and the two most important PCA components of each marker were input for CoA using the ade4 R package (Dray & Dufour, 2007). We tested the significance of the co‐relationship between matrices by comparing the CoA estimated from the empirical data set with the CoA distribution estimated after 1,000 bootstraps.

## 3 Results

### 3.1 MHC diversity and population structure

Mean MHC coverage was 635 reads (SD ± 436) per individual. Using 27 duplicate samples, the degree of change sequencing modelling provided complete allele repeatability in 22 individuals (81.5%), which is a similar rate reported in other studies (e.g. Herdegen et al., 2014). Four individuals (14.8%) had a mismatch at one allele, and one individual (3.7%) had a complete allelic mismatch. In contrast, allele repeatability was much lower for the allele validation threshold method (67%). Most of the inconsistencies in allele calling by this method were for alleles at low frequencies (<4%). We therefore based further analyses on allele calling by the degree of change sequencing modelling approach, which also effectively identified samples of low quality.

We identified 26 MHC alleles from the eight sampled regions (GenBank KU059084–KU059109). Overall polymorphism showed 23 segregating sites with average Jukes–Cantor pairwise differences of 6.4% (SD = 1.4%). Individuals had one to four MHC alleles (four alleles: 8.5%, three alleles: 31.8%, two alleles: 44.5% and one allele: 15.2%, Fig. S1). One MHC allele (H16) predominated in all sampled regions, but the presence and frequency of the other 24 alleles varied clearly among subspecies and regions. Four MHC alleles (H03, H04, H14 and H20) were detected only in *T. t. taxus*. Allele H07 occurred at the highest frequency in ON badgers followed by UP and LP, while allele H19 occurred at highest frequency in TO (Fig. 1a). *Taxidea t. taxus* badgers in AB and MB had the highest MHC allelic richness, while *T. t. jacksoni* badgers had the lowest (Table 2). Badgers from AB also had the highest number of private alleles, whereas *T. t. jacksoni* had none (Table 2). In contrast, *T. t. jeffersonii* from TO showed the highest MHC diversity within individuals (*A*_d_ **= .71), followed by *T. t. jacksoni* (*A*_d_ **= .62). Differences in values of MHC individual diversity among populations were statistically significant (*F *=* *2.42, *df* = 7, *p *=* *.02), and pairwise post hoc comparisons showed that significant differences were between TO versus AB, and TO versus MB (*p *<* *.05).

STRUCTURE analyses of MHC data identified *k *=* *3 as the most likely scenario, with the clusters corresponding roughly to subspecies designations. One cluster included *T. t. jeffersonii* from TO; the second included *T. t. taxus* from AB, SK, and MB; and the third included *T. t. jacksoni* from LP and ON. Samples from EK and UP showed admixture between their geographically adjacent clusters (Fig. 1a).

### 3.2 MHC selection and recombination test

All observed MHC alleles differed by at least one amino acid (Table S2) and were presumed functional based on lack of stop codons, an absence of frame‐shift mutations, or extremely high amino acid sequence identity (90%–96%) with functional mammalian DRB alleles (GenBank). The one‐tailed *Z*‐test of positive selection showed a nonsignificant excess of nonsynonymous substitutions for all regions, PBR and non‐PBR sites. However, the difference between the *d*_*S*_ and *d*_*N*_ ratios was larger for PBR sites compared to non‐PBRs (0.7 vs. 0.92, respectively; Table S3). The genetic algorithm recombination detection method found no significant evidence (*p *>* *.05) of recombination in the 22 potential breakpoints explored. Table 1 summarizes the individual codons identified as potentially under positive selection. Codeml identified eleven codons from three models of positive selection: M2a, M3 and M8. These models had a better fit than models without selection (M0, M1a, M7) based on LRT (Table S4). FEL identified two codons, REL three, and MEME four. From 14 codons under positive selection, eleven corresponded to PBR sites and five were identified by more than one method.

**Table 1:** Codon sites predicted to be under positive selection by four codon‐based methods of selection in 26 MHC sequences for the American badger

	Sites under positive selection													
		a	a	a	a	a	a		a	a	a	a		a
Method	1	3	5	12	22	31	32	33	42	46	49	53	55	61
FEL			X											X
REL								X					X	X
MEME							X				X	X		X
Codeml	X	X	X	X	X	X	X		X	X	X	X		

FEL, fixed effects likelihood; REL, random effects likelihood; MEME, mixed effects model of evolution.

a Putative PBR sites are based on Brown et al. (1993) and Stern et al. (1994).

### 3.3 Microsatellite diversity and population structure

We found <2% genotyping error rate, largely corresponding to ON samples that had low DNA template amounts from hairs (2–4 hairs with small roots). We detected significant departures from HWE for eight microsatellite loci (Tt22, Tt23, Mvis72, Tt‐2, Tt3, Tt‐4, Mvis87, Ma15), but with no consistent patterns across sampling regions. Most deviations from HWE were observed for TO and none in SK. Significant LD was detected for one pair of loci at two sampling regions (EK and AB: Tt15 × Tt27), but the overall data set did not show any significant LD. We retained all 20 microsatellites for subsequent analyses, as significant deviations of HWE and LD were not consistent across regions. Nineteen microsatellite loci were polymorphic in all sampling regions. The only exception was Tt‐1, which was monomorphic in ON badgers. Badgers from AB, SK and MB showed the highest values of genetic diversity (Table 2), but only *A*_r_ was statistically different (*p < *.05) between small, isolated populations relative to nonperipheral badgers. *Taxidea t. taxus* badgers showed lower inbreeding coefficients and larger estimates of effective population sizes relative to TO and ON populations (Table 3). There was a significant relationship between the mean number of alleles at MHC and microsatellites (r = .76, *p *=* *.03). We found no significant correlation between private microsatellite allelic richness and the number of private MHC alleles (r=−.02, *p *=* *.95).

**Table 2:** Estimates of genetic diversity for 20 neutral microsatellites and MHC in eight populations of American badger subspecies at the northern portion of the species range: Observed heterozygosity (*H*_o_), expected heterozygosity (*H*_e_), rarefied allelic richness (*A*_r_), rarefied private allele richness (*P*_r_), inbreeding coefficient (*F*_IS_). Number of MHC alleles (*H*), unique MHC alleles (*H*_u_), MHC individual diversity (*A*_d_), average MHC pairwise nucleotide differences (*P*_i_)

Subspecies	Location	*N*	Microsatellite loci					MHC loci			
			*h*_o_	*h*_e_	*a*_r_	*p*_r_	*f*_is_	*h*	*h*_u_	*A*_d_	*p*_i_
*T. t. jeffersonii*	TO	30	.61	.72	5.9	0.19	.17	10	0	.71	7.9
*T. t. jeffersonii*	EK	30	.68	.76	7.2	0.31	.11	14	1	.58	6.95
*T. t. taxus*	AB	39	.71	.79	7.6	0.15	.12	16	2	.54	7.39
*T. t. taxus*	SK	25	.79	.81	7.6	0.14	.01	16	1	.55	8.62
*T. t. taxus*	MB	48	.75	.81	7.9	0.24	.07	14	1	.57	8.63
*T. t. taxus*	UP	22	.6	.73	6.3	0.01	.17	10	1	.54	8.72
*T. t. jacksoni*	LP	23	.55	.7	6.3	0.26	.22	8	0	.62	5.58
*T. t. jacksoni*	ON	19	.35	.44	3.7	0.05	.21	9	0	.62	6.52

Population abbreviations: Thompson‐Okanagan (TO), East‐Kootenay (EK), Alberta (AB), Saskatchewan (SK), Manitoba (MB), Upper Peninsula (UP) and Lower Peninsula (LP) of Michigan, and Ontario (ON).

**Table 3:** Estimates of effective population size (*N*_e_) in eight sampling regions of American badgers. *N*_e_ was estimated using two methods: Linkage disequilibrium and molecular co‐ancestry with their corresponding 95% confident intervals, which are shown in parentheses. Abbreviations as in Table 2

Location	*N*	*N*_e_ (95% CI)	
		Linkage disequilibrium	Molecular co‐ancestry
TO	52	65.8 (54.5–85.8)	34 (2.5 ± 106)
EK	33	54 (43–70.7)	28.2 (6.8 ± 64.5)
AB	46	238.5 (145.2–602.8)	87.4 (1 ± 438)
SK	25	389.7 (151–∞)	∞
MB	48	∞	25.6 (10.3 ± 47.3)
UP	22	29 (23.2–37.6)	28.2 (2.1 ± 87)
LP	23	16 (13.3–19.4)	4.5 (2.8 ± 6.7)
ON	20	8.6 (5.6–13)	1.7 (1.2 ± 2.4)

STRUCTURE analysis of microsatellite data again identified *k *=* *3 clusters, but these clusters were less consistent with subspecies designations than the MHC results and suggested substantial gene flow across the borders of the subspecies’ ranges. The first cluster included badgers from TO, while EK samples (*T. t. jeffersonii*) were assigned strongly to a second cluster containing the *T. t. taxus* samples. A third cluster included the ON samples (*T. t. jacksoni),* with UP (*T. t. jacksoni)* and LP (*T. t. taxus*) badgers showing high genetic admixture between their adjacent clusters (Fig. 1b). Results from the *k *=* *5 model further differentiated EK and ON badgers (Fig. S2), suggesting that these populations have been isolated for several generations. STRUCTURE results were consistent for co‐dominant microsatellite data and binary‐encoded microsatellite data (results not shown).

**Figure 2:**
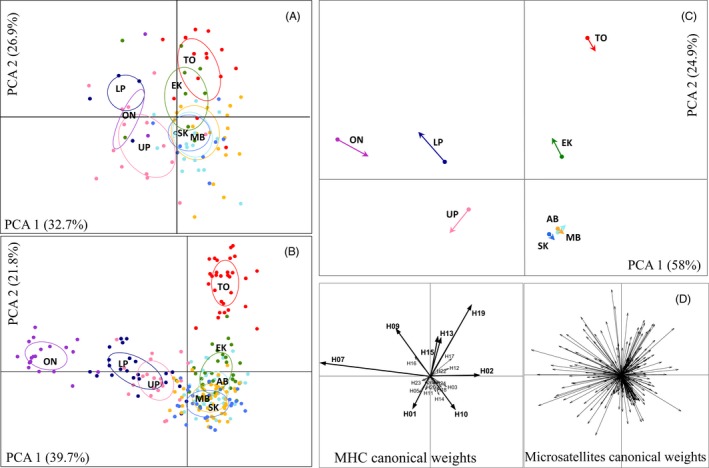
Co‐inertia analysis (CoA) between MHC and microsatellite data for eight populations. Ordination of first two factorial PCA axes for (A) MHC and (B) microsatellite loci, where dots represent individuals within sampling regions distinguished in different colours. (C) CoA plot, sho1wing the relative position of each population on the factorial plane for the first two CoA eigenvalues. The dots represent the variation observed at microsatellites, while the arrows represent the variation at MHC. The length and direction of the vector denote the translational coefficient of the population position relative to each other, while the strength of the correlation between microsatellite and MHC data sets for each sampling region is inversely correlated with the vector length; (D) bottom figures represent canonical weights for MHC and microsatellites.

### 3.4 Comparisons between neutral and functional diversity

AMOVAs showed that the proportion of genetic variance partitioned among sampling regions was higher for MHC (18%, *p *<* *.05) than microsatellites (10%, *p *<* *.05). AMOVA based on the three subspecies showed a similar trend, but explained a lower proportion of the genetic variance compared to the grouping by sampling region (MHC: subspecies=11%, *p *<* *.05; sampling regions within subspecies = 10%, *p *<* *.05; microsatellites: subspecies = 6%, *p *<* *.05; sampling regions within subspecies = 6%, *p *<* *.05).

Factorial PCA based on MHC data did not identify clear genetic differentiation among subspecies or sampling regions (Fig. 2a), but factorial PCA based on microsatellites confirmed the strong genetic differentiation of TO and ON from the other regions (Fig. 2b). The global co‐inertia coefficient revealed a significant positive correlation between MHC and microsatellite variation in the full data set (RV‐coefficient = .87, *p *=* *.0001), but the strength of this correlation varied among regions (for example, AB, MB and SK showed the shortest vector length and thus the highest correlation). The CoA plot shows the joint trend of the covariance between the MHC and microsatellites for each region (Fig. 2c). The vector length is inversely proportional to the covariance between data sets; that is, the longer the length the higher the divergence between markers. MHC canonical weights indicated that alleles H07 and H09 contributed largely to the separation of ON and LP badgers; alleles H15, H13 and H19 contributed to the discrimination of TO and EK badgers; allele H10 to the separation of MB, AB and SK; and allele H01 contributed to the discrimination of UP badgers (Fig. 2c). These alleles occurred with the highest frequencies in each of these sampling regions (Fig. 1).

Pairwise *F*_ST_ values for both microsatellites and MHC supported the genetic differentiation of TO and ON badgers from other populations (Table S5). Mantel test showed a positive significant correlation of geographical and genetic distances for MHC (*M*_r_ = .71, *p *=* *.002) and microsatellites (*M*_r_ **= .53, *p *=* *.01). Controlling for the effect of neutral genetic differentiation on MHC against geographical distances in the partial Mantel test did not change observed trends (*M*_r_ **= .6, *p *=* *.007). MHC and microsatellite genetic distances were significantly correlated (*M*_r_ **= .49, *p *=* *.015).

## 4 Discussion

Our results show the importance of neutral processes in shaping the distribution of MHC polymorphism across the northern range of the American badger and highlight the stronger interactions between gene flow, genetic drift and selection towards the western and eastern peripheries of the badger range. The strong similarity of MHC alleles within designated subspecies relative to their neutral genetic differentiation indicates adaptive divergence among subspecies. This suggests that subspecies can be considered ecomorphs, which respond to different selective pressures across their range through local adaptation. These results can better inform conservation management of endangered badger populations, by shifting the focus of conservation efforts towards the conservation of adaptive genetic diversity across the species range.

### 4.1 MHC allele variation and positive selection

Major histocompatibility complex DRB exon 2 diversity in the American badger was moderate relative to other species. Badger MHC diversity (26 alleles in 236 individuals) was similar to the Finnish wolf (*Canis lupus*, Niskanen et al., 2014) and the montane vole (*Microtus montanus*, Winternitz & Wares, 2013), higher than in the European badger (*Meles meles*, Sin, Dugdale, Newman, Macdonald, & Burke, 2012), wolverines (*Gulo gulo*, Rico et al., 2015), the European mink (*Mustela lutreola*, Becker, Nieberg, Jahreis, & Peters, 2009) and sea otters (*Enhydra lutris*, Aguilar, Jessup, Estes, & Garza, 2008), but lower than in raccoons (*Procyon lotor*, Kyle et al., 2014) and the brown bear (*Ursus arctos*, Kuduk et al., 2012). Moreover, the occurrence of one to four alleles within individuals suggests the presence of two MHC DRB exon 2‐like loci. Copy number variation is a common feature in many vertebrates (e.g. Figueroa et al., 2001; Van Oosterhout et al., 2006; Oomen et al., 2013; Kyle et al., 2014; Lighten et al., 2014), which is explained by gene duplications from a birth–death process where some duplicated genes are maintained by balancing selection for a long time, while others are eliminated or become nonfunctional (Nei, Gu, & Sitnikova, 1997; Axtner and Sommer 2007).

Evidence of positive selection at MHC was not detected using a one‐tailed *Z*‐test for all sequences, nor on putative PBR, although the average of nonsynonymous to synonymous substitutions was higher in PBR sites. However, maximum‐likelihood methods detected 14 of 61 codons under significant positive selection, most of which corresponded to PBRs. These results agree with a large number of studies that show balancing selection on MHC PBR sites (e.g. Biedrzycka & Radwan, 2008; Luo et al., 2012). However, maximum‐likelihood methods detect historical periods of balancing selection during the evolutionary trajectory of a species (Garrigan & Hedrick, 2003). Signatures of selection acting across contemporary populations are thus better evaluated by contrasting genetic differentiation at neutral and MHC loci (Ekblom et al., 2007)

### 4.2 Patterns of genetic structure in neutral and MHC loci

For both markers and consistently among analyses, we observed clear genetic differentiation of TO and ON badgers from other sampling regions. The increasing genetic differentiation towards the periphery was in agreement with previous studies using microsatellites (Kyle et al., 2004) and mtDNA (Ethier et al., 2012). Based on mtDNA, there was clear genetic differentiation between EK and *T. t. taxus*, whereas UP was genetically more similar to *T. t. taxus* than to *T. t. jacksoni,* and thus, UP badgers were re‐designated as *T. t. taxus* (Ethier et al., 2012). In contrast, our microsatellite data showed much weaker genetic structuring along the boundaries of the recognized subspecies, which suggest substantial gene flow among them, and sex‐biased dispersal.

In the west, the neutral genetic insularity of TO badgers can be explained by limited dispersal imposed by the rugged topography of the Selkirk Mountains between TO and EK regions. Likewise, the lower quality habitat (i.e. rocky clay soils and low prey availability) between TO and EK, and the Flathead Montana and north‐west Washington is also expected to limit population connectivity (COSEWIC, 2012). In the east, the increasing genetic differentiation of ON badgers is likely the result of historical hydrological barriers to dispersal such as the Great Lakes between Ontario and Michigan, and contemporary habitat fragmentation due to land‐use changes (agricultural intensification, settlements, road density). The observed neutral genetic admixture of EK and *T. t. taxus* suggests that gene flow between them is more substantial than previously anticipated. Lack of genetic substructure for the prairie *T. t. taxus* badgers at both markers, but differentiation of UP badgers might indicate that *T. t. taxus* is not panmictic across its range. This result needs to be interpreted with caution as our UP samples were from the edge of its northern distribution (Fig. 1), and samples between MB and central United Sates need to be included to determine whether the observed genetic differentiation is detectable along the *T. t. taxus* range.

The similarity of MHC alleles between populations within subspecies, despite their geographical isolation towards the range margins, might reflect high effective migration rates of beneficial MHC alleles (McMullan & Van Oosterhout, 2012). The higher similarity at MHC between these populations was shown in CoA, where particular MHC alleles appear to drive their divergence. Empirical studies have shown that varying frequencies of MHC alleles can result from their association with susceptibility to infections (e.g. Meyer‐Lucht & Sommer, 2005; Sin et al., 2012; Srithayakumar, Castillo, Rosatte, & Kyle, 2011). Under balancing selection, population differentiation at MHC is expected to be weaker compared to neutral loci as advantageous alleles are selected for despite reduced gene flow between populations (Schierup, Vekemans, & Charlesworth, 2000). CoA also revealed a smaller correlation of MHC and neutral loci in *T. t. jeffersonii* and *T. t. jacksoni* populations compared to the larger correlation for the Prairie badgers (e.g. smaller vector length). This result indicates a larger discrepancy in the degree of joint genetic structure between MHC and neutral loci towards the peripheries, suggesting ecological adaptation.

The American badger is an omnivore across its range, feeding on most available small mammals, reptiles, birds and invertebrates (Azevedo et al., 2006). Variation in badger diet across Canada corresponds with a shift in community structure of prey species from east to west. Eastern badgers are sympatric with small mammal communities dominated by eastern chipmumk (*Tamius striatus*), mice (*Peromyscus* sp.), woodchucks (*Marmota monax*) and eastern cottontail (*Sylvilagus floridanus*) (Dobbyn, 1994). In the west, small mammal communities are dominated by black‐tailed prairie dogs (*Cynomys ludovicianus*), ground squirrels (*Spermophilus* sp.) and marmots (*M. flaviventris, M. caligata;* Michener, 2000; Kinley & Newhouse, 2008; COSEWIC, 2012). Thus, differences in species consumed by badgers across its northern range may expose them to a different suite of pathogens. Western populations are exposed to *Yersinia pestis,* the bacterium responsible for causing plague. The pathogen is maintained in populations of ground squirrels and prairie dogs that are important prey items for western badgers. *Yersinia pestis* can persist in carcasses and surrounding soil for up to 7 months, and badgers frequently cache prey for weeks prior to consumption (Michener, 2000). Moreover, evidence of exposure to canine distemper, canine parvovirus and leptospirosis have been found in ON badgers (D.M. Ethier, J.B. Sayers, C.J. Kyle, C.J. Nocera, & D. Campbell, unpublished manuscript). Other pathogens likely affecting badgers across Canada include rhabdoviruses, *Trichinella* species and *Franciscella tularensis,* which cause rabies, trichinellosis and tularaemia (COSEWIC, 2012). Associations between MHC variation and specific pathogens could be addressed in future by overlapping MHC genotypes with comprehensive pathogen screening of badgers across their range.

While selection by pathogens is a likely driver of MHC diversity in badgers, we cannot rule out the contribution of gene flow in shaping MHC differentiation, as we found a significant pattern of isolation by distance for MHC and neutral loci, and a positive correlation between MHC and microsatellite genetic distances. Landscape genetic analysis together with computer simulations could be applied in future in this system to test the relative influence of geographical isolation and major landscape features such as the Selkirk Mountains and the Great Lakes in determining spatial genetic structure.

### 4.3 Levels of diversity in neutral and MHC loci

We found lower neutral genetic diversity in small, peripheral populations relative to nonperipheral populations with significant differences for allelic richness. Similarly, the number of MHC alleles was higher in large compared to small populations, but MHC diversity within individuals was not lower in small populations. Levels of genetic diversity are expected to respond to demographic processes such as inbreeding, genetic drift, restricted gene flow and small population size (Frankham, 2005). The significant correlation between the number of MHC and microsatellite alleles suggests effects of demographic processes influencing neutral and MHC genetic diversity. Furthermore, estimates of population size suggest that fewer than 200 individuals occur in ON, and as few as 150 and 250 individuals persist in EK and TO, respectively (COSEWIC, 2012). The population size of *T. t. taxus* in the Canadian Prairies is much larger with an estimated 1,000–10,000 individuals (Scobie, 2002). We observed in small populations higher *F*_IS_ coefficients and smaller *N*_e_ estimates relative to large populations, which partly can explain the differences in genetic diversity found between small and large badger populations. However, the higher MHC diversity within individuals in small populations can be instead associated with selective pressures. This suggests that despite small *N*_e_, genetic drift might not be strong enough to offset the strength of selection and erode adaptive genetic variation in small, isolated badger populations.

Similar empirical studies in mammalian endangered species have reported low MHC diversity in cheetahs (*Acinonyx jubatus*, Castro‐Prieto et al., 2012), Tasmania devil (*Sarcophilus harrisii*, Morris, Austin, & Belov, 2013), European bison (*Bison bonasus*, Radwan, Kawałko, Wójcik, & Babik, 2007), giant panda (*Ailuropoda melanoleuca*, Zhang, Wu, Hu, Wu, & Wei, 2015), mountain goat (*Oreamnos americanus*, Shafer, Fan, Côté, & Coltman, 2012), and the golden snub‐nosed monkey (*Phinopithecus roxellana*, Luo et al., 2012), but there are notable exceptions. For instance, balancing selection instead of genetic drift explained high MHC polymorphism in bottlenecked populations of the Finnish wolf (*Canis lupus*, Niskanen et al., 2014) and in the Island fox (*Urocyon littoralis*, Aguilar et al., 2004).

Several mechanisms have been proposed for the erosion of MHC diversity in small populations. The most common explanation suggests that genetic drift might outweigh the strength of selection, resulting in a reduction of MHC alleles at the population level and loss of copy number variation within individuals, either by stochastic removal of haplotypes with multiple gene copies or by fixation of the same allele at multiple loci (Eimes et al., 2011). Rare MHC alleles could be lost more rapidly than common alleles if populations have a skewed allele distribution resulting from negative frequency‐dependent selection (Sutton, Nakagawa, Robertson, & Jamieson, 2011). Alternatively, in small populations, even when genetic drift would otherwise erode MHC diversity, MHC high copy number variation within individuals can be selected for through heterozygote advantage if high allele variation allows broader recognition of pathogens (Piertney & Oliver, 2006; Niskanen et al., 2014; Savage and Zamudio 2016). Our results are consistent with this latter hypothesis. A third possibility is that small populations with reduced genetic variation can retain MHC alleles with high levels of divergence (Hedrick, 2002; Lenz, 2011), although evidence supporting this premise is limited (see Ejsmond, Babik, & Radwan, 2010).

### 4.4 Conservation implications

Our findings show that subspecies designations are more congruent with the functional genetic clusters than with the neutral microsatellite data. Thus, subspecies may be more appropriately considered as ecotypes such as those observed in grey wolves (*Canis lupus*; Leonard, 2015) or Grizzly Bears (*Ursus arctos*, Shafer, Nielsen, Northrup, & Stenhouse, 2014). Recognizing adaptive divergence is critical for prioritizing which populations to protect and for selecting the best population sources for translocations or assisted migration (Funk, McKay, Hohenlohe, & Allendorf, 2012; Latta, 2008). For conservation and management purposes, the Canadian government independently assesses four designatable units of badgers on the basis of population size estimates and neutral genetic data (COSEWIC, 2012; TO and EK *T. t. jeffersonii* populations were separated because of their geographical isolation and neutral genetic differentiation). Our data support these divisions and can greatly inform conservation efforts. For example, if only microsatellite data is used, the designation of TO and ON as distinctive conservation units would be supported, but the indication of substantial gene flow between EK and *T. t. taxus* populations would not warrant their designation as different units. On the other hand, the MHC genetic differentiation of EK from *T. t. taxus* and its higher MHC similarity with TO likely suggest that EK badgers are under similar selective pressures to the badgers in TO. Therefore, translocating EK and *T. t. taxus* badgers into each other's environments might increase the probability of outbreeding depression resulting from the reproduction between adaptively diverged populations (Frankham et al., 2011).

Although MHC loci capture a fraction of the genetic variation underpinning resistance to pathogens (Acevedo‐Whitehouse & Cunningham, 2006), evidence from several species have shown that the MHC is useful in examining the adaptive potential of populations in mammalian species (e.g. Aguilar et al., 2004; de Assunção‐Franco, Hoffmam, Harwood, & Amos, 2012; Schweizer et al., 2016; Siddle, Marzec, Cheng, Jones, & Belov, 2010), and for informing delineation of conservation units in endangered species such as the giant panda (*Ailuropoda melanoleuca,* Zhu, Wan, Yu, Ge, & Fang, 2013) and marbled murrets (*Brachyramphus marmoratus,* Vásquez‐Carrillo, Friesen, Hall, & Peery, 2014). Conservation efforts should ideally focus on maximizing the species evolutionary potential and thus should gather genetic data from multiple functional loci (Grueber, 2015). More comprehensive approaches that utilize hundreds of genetic markers (e.g. single nucleotide polymorphism, SNPs) to examine functional variation in multiple immunity genes through genome‐sequence data, would improve our understanding on how wildlife populations respond to disease (Morris et al., 2013). On the other hand, adaptive divergence revealed through a single‐locus such as the MHC might not reflect divergence in adaptive genes associated with other relevant ecological conditions (Zhou, Seim, & Gladyshev, 2015; Schweizer et al., 2016). Screening for genome‐wide variation to identify putative loci under selection or associations between genetic variants and phenotypic traits is the plausible option, which is increasingly being used to investigate adaptive genetic variation in wildlife (Harrisson, Pavlova, Telonis‐Scott, & Sunnucks, 2014; Schweizer et al., 2016).

Evaluating evolutionary potential in wildlife populations is still challenging (Allendorf, Hohenlohe, & Luikart, 2010). For instance, many traits that are currently adaptive may not confer fitness under the rapid ongoing environmental pressures (Funk et al., 2012). Moreover, many complex phenotypic traits are known to be polygenic, which implies that single‐locus methods become insufficient as the number of loci involved can be large (Le Corre & Kremer, 2012). Other challenges include aspects of statistical analysis, where most available methods are prone to large number of false discoveries for trait‐associations from genome‐wide data. Despite these existing challenges, genomic approaches could ideally provide more comprehensive foundations to move towards an adaptive species management (Harrisson et al., 2014).

Badgers face many threats across their range as habitat is becoming increasingly fragmented by land‐use changes. Areas of high urbanization and road density are expected to drastically limit badger movements and increase incidental deaths by road crossing (COSEWIC, 2012). At the same time, encroachment of forest into native grasslands in British Columbia may limit the size and carrying capacity of remaining habitat. These landscape modifications limit gene flow among the increasingly isolated populations. The resulting increase in genetic drift and inbreeding and the concurrent impacts on adaptive genetic diversity can be mitigated in part by restoration and protection of badger habitat. Other threats faced by *T. taxus* in Canada are either poorly documented (for example, the magnitude of mortality from disease) or require a more direct approach to mitigation (i.e. human persecution). For example, badgers are poisoned either secondarily, due to consumption of prey that have consumed rodenticides, or directly in cases where landowners do not want badgers on their property (COSEWIC, 2012; Proulx and MacKenzie 2012). Diseases such as plague, leptospirosis and canine distemper can also increase mortality of adults (Quinn et al., 2012), with particularly strong effects on small and isolated populations.

Habitat loss and climate change are expected to promote the expansion of pathogens and their vectors and thus increase the incidence of diseases affecting wildlife in northern regions (Brooks & Hoberg, 2007; Kutz et al., 2009). The capacity of wildlife populations to persist under such conditions is a critical topic in conservation biology (Berteaux, Réale, McAdam, & Boutin, 2004; Altizer, Ostfeld, Johnson, Kutz, & Harvell, 2013; Hoberg and Brooks 2015). Examining the spatial distribution of MHC polymorphism among populations is a valuable proxy for understanding the processes underpinning their evolutionary potential (Radwan, Biedrzycka, & Babik, 2010; Sommer, 2005), which is fundamental to better inform conservation strategies. Our study thus underscores the importance of considering neutral and functional genetic markers to simultaneously evaluate the adaptive genetic potential of small and isolated populations in mammalian species (Frankham, 2010).

## Data Archiving

Microsatellite genotypes at 20 loci and binary‐encoded data of MHC DRB exon 2 for 236 samples of American badgers are available on Dryad: doi:10.5061/dryad.qb87r. MHC sequences can be accessed on GenBank KU059084‐KU059109.
