# Supplementary material for: Spatial patterns of immunogenetic and neutral variation underscore the conservation value of small, isolated American badger populations
Source: Evol Appl. 2016 Aug 21;9(10):1271–84. doi: 10.1111/eva.12410 (PMC5108218; doi:10.1111/eva.12410)

S1. Frequency distribution of MHC alleles per individual within the eight sampling regions of the American badger (*Taxidea taxus*)

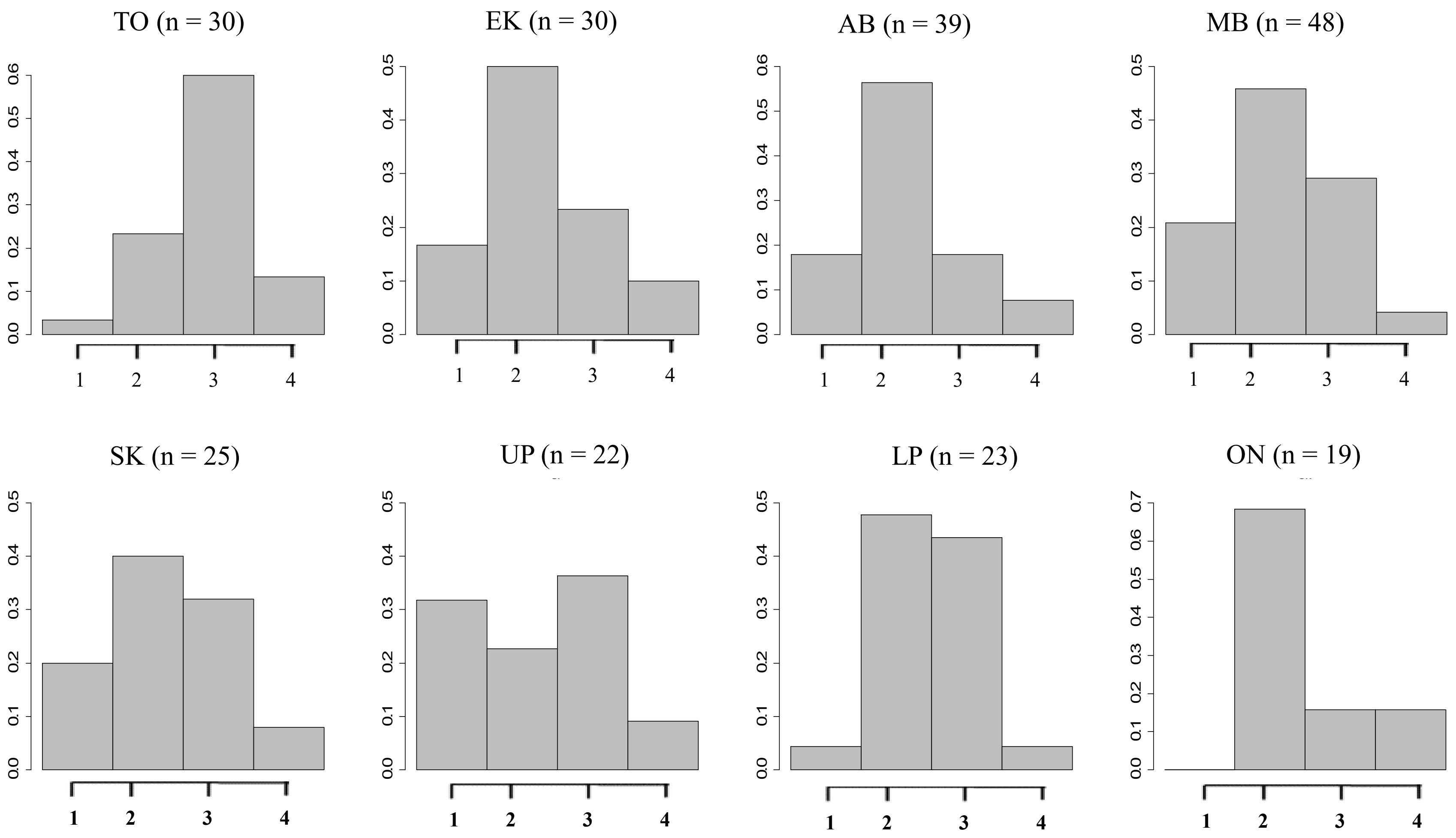

Supplement: Supplementary file 1 [file EVA-9-1271-s001.pdf]
