# Supplementary material for: Spatial patterns of immunogenetic and neutral variation underscore the conservation value of small, isolated American badger populations
Source: Evol Appl. 2016 Aug 21;9(10):1271–84. doi: 10.1111/eva.12410 (PMC5108218; doi:10.1111/eva.12410)

Fig S2. Structure *k* =5 plot for binary-encoded data of 20 microsatellite loci in eight sampling populations of the American badger (*Taxidea taxus*)

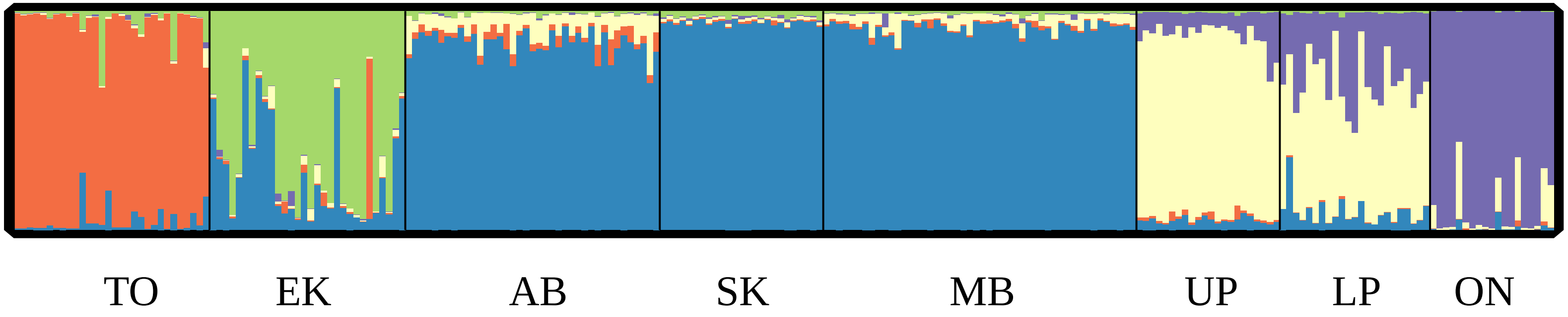

Supplement: Supplementary file 2 [file EVA-9-1271-s002.pdf]
