# Supplementary material for: Spatial patterns of immunogenetic and neutral variation underscore the conservation value of small, isolated American badger populations
Source: Evol Appl. 2016 Aug 21;9(10):1271–84. doi: 10.1111/eva.12410 (PMC5108218; doi:10.1111/eva.12410)
Supplement: Supplementary file 3 [file EVA-9-1271-s003.pdf]

Table S1. Primer sequences and characteristics of 20 polymorphic microsatellite markers amplified in three subspecies of North American badgers (*Taxidea taxus*)

Multiplex	Locus	Label	Range (bp)	Motif	Ta (◦C)	Sequence Forward	Sequence Reverse	Alleles	Reference
A	Mvis072	FAM	260-310	$(CA)_{15}$	58	CTGCAAAGCTTAGGAATGGAGA	CCACTACACTGGAGTTTCAG[illegible]	8	Flemming et.al. 1999
	Tt21	FAM	194-212	$(AC)_{17}$		TACCAAACTTGTATCCCATGTAG	GGTCACCAAGATACGAATGA	9	Rico et al. (2014)
	Tt27	FAM	148-170	$(AC)_{17}$		AGAGTCCAGATATCCATTGACAG	ACATCATTGCAAATACAACA[illegible]	11	Rico et al. (2014)
	Gg465	HEX	160-185	$(CA)_{20}$		GATCTTCACAAACAAGCTTC	GATCTCCTTTCCTCTCTTTG	13	Walker etal. 2001
	Ggu443	NED	80-110	$(CA)_{14}$		GATCATGTTTGCAATTAAATGT	GATCCTCCGGTAACTGTTGT	14	Walker et al. 2001
B	Tt15	FAM	168-188	$(GT)_{18}$	60	TGAGAAGCCTGACTTGGAGCA	GAGGTTGTCTGCAACTAGCA[illegible]	14	Rico et al. (2014)
	Tt3	HEX	158-176	$(TG)_{23}$		AGTCTGCTTGGGATTTTCTC	TTTTGCTCTATGATTACACC	13	Davis & Strobeck. 1998
	Tt4	NED	172-188	$(TG)_{18}$		GGTGAGACCCTGGAAATAGAAA	GCTAACCAAACCTAGCAATG[illegible]	13	Davis & Strobeck. 1998
	Tt20	NED	119-143	$(AC)_{17}$		TGCAGCCAAACAGTAACGAC	GTCGACATGTATGCTGCATG[illegible]	12	Rico et al. (2014)
C	Mvi87	HEX	81-113	NA		AACAATAGTAGTGGCAGCAGC	GTCTGTGAAACACTGCAAAG[illegible]	12	O'Connell et al 1996
	Tt13	FAM	182-204	$(GT)_{19}$	54	CAGCATTGGGCAACTGGAC	TTTCCATAACTAGAGGTACT[illegible]	13	Rico et al. (2014)
	Tt1	NED	152-172	$(TG)_{20}$		AACGGCTTCTAACCACTCCA	CCCCGCTTTTCATTTCTTTA	13	Davis & Strobeck. 1998
	Tt2	HEX	197-213	$(TG)_{11}$		AGCCAAGACACAGAAACAAC	TTCAAGGATTCAAGGACCAT	7	Davis and Strobeck, 1998
D	Ggu234	FAM	92-102	$(CA)_{25}$	50	TTACTTAGAGGATGATAACTTG	GAACTCATAGGACTGATAGC	9	Duffy et al. 1998
	Tt23	FAM	145-165	$(AC)_{17}$		GGGATACCAATTAGAATCCACA	CAAAGATCACTGTGATGGTG[illegible]	9	Rico et al. (2014)
	Tt17	HEX	168-188	$(GT)_{18}$		ATTGGCTGTTAGAGTCAGGAATC	ACAAGCTGTTACTTCACAAC[illegible]	11	Rico et al. (2014)
	Ma15	HEX	310-335	$(TG)_{9}$		GGAAATGGTCCACAGTCTTAG	CCAGTAAGGCCAAATATCAA[illegible]	12	Davis & Strobeck. 1998
E	Ma1	NED	192-204	$(TG\text{-}TG)_{19}$	54	ATTTTATGTGCCTGGGTCTA	TTATGCGTCTCTGTTTGTCA	11	Davis & Strobeck. 1998
	MP0085	HEX	150-200	$(TG)_{12}$		AGGGAGTCTCTGCAGGTGAA	ACAATTGGTGTCAGGCAATG	5	Jordan et al. 2007
	Tt22	FAM	122-130	$(AC)_{17}$		CTCAAATGAATGTTCAAGGCTC	AGAACACTGGTCTGTGGTTC[illegible]	9	Rico et al. (2014)
