# Supplementary material for: Spatial patterns of immunogenetic and neutral variation underscore the conservation value of small, isolated American badger populations
Source: Evol Appl. 2016 Aug 21;9(10):1271–84. doi: 10.1111/eva.12410 (PMC5108218; doi:10.1111/eva.12410)
Supplement: Supplementary file 4 [file EVA-9-1271-s004.pdf]

Table S2. Aminoacid sequences of the Major Histocompatibility Complex (MHC) DRB exon 2 in the American badger (*Taxidea taxus*).

Dots denotes sequence identity to the top allele. Putative PBR sites (*) are based on Brown et al. (1993) and Stern et al. (1994)

**Allele**										*		*		*					*	*									*									*	*			*	*				*		*	*		*	*			*				*			*	*			*	*							
KU059109	N	G	T	E	R	V	R	L	L	**D**	R	**Y**	F	**Y**	N	G	E	E	**Y**	**V**	R	F	D	S	D	V	G	E	**Y**	R	P	V	T	E	L	G	R	**R**	**T**	A	E	**Y**	**W**	N	S	Q	**K**	D	**I**	**L**	E	**R**	**T**	R	A	**E**	V	D	T	**Y**	C	R	**H**	**N**	Y	G	**V**	**V**	E	S	F	T	V	Q	R
KU059108	.	.	.	.	.	.	.	.	.	E	.	Q	.	.	.	.	.	.	.	.	.	.	.	.	.	.	.	.	F	.	.	.	.	.	.	.	.	P	D	.	.	.	.	.	.	.	.	.	.	.	.	Q	K	.	.	.	.	.	.	V	.	.	.	.	.	.	.	G	.	.	.	.	.	.	.
KU059107	.	.	.	.	.	.	.	F	.	E	.	Q	.	.	.	.	.	.	V	.	.	.	.	.	.	.	.	.	.	.	.	.	.	.	.	.	.	P	D	.	.	.	.	.	.	.	.	.	.	.	.	Q	K	.	.	N	.	.	.	.	.	.	.	.	.	.	.	G	.	.	.	.	.	.	.
KU059106	.	.	.	.	.	.	.	.	.	.	.	.	.	.	.	.	.	.	H	.	.	.	.	.	.	.	.	.	F	.	.	.	.	.	.	.	.	P	D	.	.	.	.	.	.	.	.	.	.	.	.	Q	K	.	.	N	.	.	.	.	.	.	.	.	.	.	.	G	.	.	.	.	.	.	.
KU059105	.	.	.	.	.	.	.	.	.	.	.	.	.	.	.	.	.	.	H	.	.	.	.	.	.	.	.	.	F	.	.	.	.	.	.	.	.	P	D	.	.	.	.	.	.	.	.	.	.	.	.	Q	R	.	.	.	.	.	.	V	.	.	.	.	.	.	.	.	.	.	.	.	.	.	.
KU059104	.	.	.	.	.	.	.	F	.	E	.	Q	.	.	.	.	.	.	H	.	.	.	.	.	.	.	.	.	.	.	.	.	.	.	.	.	.	P	D	.	.	.	.	.	.	.	.	.	.	.	.	Q	K	.	.	.	.	.	.	V	.	.	.	.	.	.	.	G	.	.	.	.	.	.	.
KU059103	.	.	.	.	.	.	.	.	.	.	.	.	.	.	.	.	.	.	.	.	.	.	.	.	.	.	.	.	F	.	.	.	.	.	.	.	.	.	.	.	.	.	.	.	.	.	.	.	.	.	.	Q	K	.	.	.	.	.	.	V	.	.	.	.	.	.	.	.	.	.	.	.	.	.	.
KU059102	.	.	.	.	.	.	.	F	.	K	.	Q	.	.	.	.	.	.	V	.	.	.	.	.	.	.	.	.	.	.	.	.	.	.	.	.	.	P	D	.	.	.	.	.	.	.	.	.	L	.	.	Q	R	.	.	.	.	.	.	.	.	.	.	.	.	.	.	.	.	.	.	.	.	.	.
KU059101	.	.	.	.	.	.	.	F	.	.	.	.	.	.	.	.	.	.	.	.	.	.	.	.	.	.	.	.	.	.	.	.	.	.	.	.	.	.	.	.	.	.	.	.	.	.	.	.	.	.	.	.	.	.	.	.	.	.	.	.	.	.	.	.	.	.	.	.	.	.	.	.	.	.	.
KU059100	.	.	.	.	.	.	.	F	.	.	.	.	.	.	.	.	.	.	.	.	.	.	.	.	.	.	.	.	.	.	.	.	.	.	.	.	.	.	.	.	.	.	.	.	.	.	.	.	L	.	.	.	.	.	.	.	.	.	.	.	.	.	.	.	.	.	.	G	.	.	.	.	.	.	.
KU059099	.	.	.	.	.	.	.	F	.	E	.	Q	.	.	.	.	.	.	H	.	.	.	.	.	.	.	.	.	.	.	.	.	.	.	.	.	.	P	D	.	.	.	.	.	.	.	.	.	.	.	.	Q	K	.	.	N	.	.	.	.	.	.	.	.	.	.	.	G	.	.	.	.	.	.	.
KU059098	.	.	.	.	.	.	.	F	.	E	.	Q	.	.	.	.	.	.	H	.	.	.	.	.	.	.	.	.	F	.	.	.	.	.	.	.	.	P	D	.	.	.	.	.	.	.	.	.	.	.	.	Q	K	.	.	N	.	.	.	.	.	.	.	.	.	.	.	G	.	.	.	.	.	.	.
KU059097	.	.	.	.	.	.	.	F	.	E	.	Q	.	.	.	.	.	.	F	.	.	.	.	.	.	.	.	.	.	.	.	.	.	.	.	.	.	P	D	.	.	.	.	.	.	.	.	.	.	.	.	Q	K	.	.	N	.	.	.	.	.	.	.	.	.	.	.	G	.	.	.	.	.	.	.
KU059096	.	.	.	.	.	.	.	F	.	K	.	Q	.	.	.	.	.	.	V	.	.	.	.	.	.	.	.	.	F	.	.	.	.	.	.	.	.	P	D	.	.	.	.	.	.	.	.	.	L	.	.	Q	K	.	.	N	.	.	.	.	.	.	.	.	.	.	.	G	.	.	.	.	.	.	.
KU059095	.	.	.	.	.	.	.	F	.	E	.	H	.	.	.	.	.	.	H	.	.	.	.	.	.	.	.	.	.	.	.	.	.	.	.	.	.	P	D	.	.	.	.	.	.	.	.	.	.	.	.	Q	K	.	.	N	.	.	.	.	.	.	.	.	.	.	.	G	.	.	.	.	.	.	.
KU059094	.	.	.	.	.	.	.	F	.	E	.	Q	.	.	.	.	.	.	H	.	.	.	.	.	.	.	.	.	.	.	.	.	.	.	.	.	.	.	.	.	.	.	.	.	.	.	.	.	.	.	.	Q	K	.	.	N	.	.	.	.	.	.	.	.	.	.	.	G	.	.	.	.	.	.	.
KU059093	.	.	.	.	.	.	.	F	.	E	.	H	.	.	.	.	.	.	.	.	.	.	.	.	.	.	.	.	.	.	.	.	.	.	.	.	.	.	.	.	.	.	.	.	.	.	.	.	.	.	.	Q	K	.	.	N	.	.	.	.	.	.	.	.	.	.	.	G	.	.	.	.	.	.	.
KU059092	.	.	.	.	.	.	.	F	.	E	.	H	.	.	.	.	.	.	H	.	.	.	.	.	.	.	.	.	.	.	.	.	.	.	.	.	.	.	.	.	.	.	.	.	.	.	.	.	.	.	.	Q	K	.	.	N	.	.	.	.	.	.	.	.	.	.	.	G	.	.	.	.	.	.	.
KU059091	.	.	.	.	.	.	.	F	.	.	.	.	.	.	.	.	.	.	.	.	.	.	.	.	.	.	.	.	.	.	.	.	.	.	.	.	.	P	D	.	.	.	.	.	.	.	.	.	.	.	.	Q	K	.	.	N	.	.	.	.	.	.	.	.	.	.	.	G	.	.	.	.	.	.	.
KU059090	.	.	.	.	.	.	.	F	.	.	.	.	.	.	.	.	.	.	.	.	.	.	.	.	.	.	.	.	.	.	.	.	.	.	.	.	.	.	.	.	.	.	.	.	.	.	.	.	.	.	.	Q	K	.	.	N	.	.	.	.	.	.	.	.	.	.	.	G	.	.	.	.	.	.	.
KU059089	.	.	.	.	.	.	.	F	.	.	.	.	.	.	.	.	.	.	F	.	.	.	.	.	.	.	.	.	.	.	.	.	.	.	.	.	.	.	D	.	.	.	.	.	.	.	.	.	L	.	.	.	.	.	.	.	.	.	.	.	.	.	.	.	.	.	.	G	.	.	.	.	.	.	.
KU059088	.	.	.	.	.	.	.	.	.	.	.	.	.	.	.	.	.	.	.	.	.	.	.	.	.	.	.	.	.	.	.	.	.	.	.	.	.	.	.	.	.	.	.	.	.	.	.	.	.	.	.	Q	K	.	.	.	.	.	.	V	.	.	.	.	.	.	.	.	.	.	.	.	.	.	.
KU059087	.	.	.	.	.	.	.	F	.	.	.	.	.	.	.	.	.	.	F	.	.	.	.	.	.	.	.	.	.	.	.	.	.	.	.	.	.	P	D	.	.	.	.	.	.	.	.	.	L	.	.	Q	R	.	.	.	.	.	.	V	.	.	.	.	.	.	.	.	.	.	.	.	.	.	.
KU059086	.	.	.	.	.	.	.	F	.	E	.	Q	.	.	.	.	.	.	H	.	.	.	.	.	.	.	.	.	.	.	.	.	.	.	.	.	.	P	D	.	.	.	.	.	.	.	.	.	L	.	.	.	.	.	.	.	.	.	.	.	.	.	.	.	.	.	.	G	.	.	.	.	.	.	.
KU059085	.	.	.	.	.	.	.	.	.	E	.	Q	.	.	.	.	.	.	H	.	.	.	.	.	.	.	.	.	.	.	.	.	.	.	.	.	.	P	D	.	.	.	.	.	.	.	.	.	L	.	.	Q	R	.	.	.	.	.	.	V	.	.	.	.	.	.	.	.	.	.	.	.	.	.	.
KU059084	.	.	.	.	.	.	.	.	.	.	.	.	.	.	.	.	.	.	H	.	.	.	.	.	.	.	.	.	F	.	.	.	.	.	.	.	.	P	D	.	.	.	.	.	.	.	.	.	.	.	.	Q	K	.	.	.	.	.	.	V	.	.	.	.	.	.	.	G	.	.	.	.	.	.	.
