# Supplementary material for: Spatial patterns of immunogenetic and neutral variation underscore the conservation value of small, isolated American badger populations
Source: Evol Appl. 2016 Aug 21;9(10):1271–84. doi: 10.1111/eva.12410 (PMC5108218; doi:10.1111/eva.12410)
Supplement: Supplementary file 5 [file EVA-9-1271-s005.pdf]

Table S3. Average rates of synonymous substitutions per synonymous site (dS) and nonsynonymous substitutions per synonymous site (dN) in percentages and standard deviation in parentheses, and Z-tests of positive selection on all sites, peptide binding region (PBR) inferred from Brown et al. (1993), and the non-PBR for the American badger (*Taxidea taxus*) MHC.

**Type**	**dS**	**dN**	**Z**	**P-value**
All	4.4 (2.6)	6.5 (2.8)	1.58	0.059
PBR	5.6 (5.2)	8.9 (4.9)	0.71	0.23
Non-PBR	4.0 (2.9)	5.5 (2.5)	0.92	0.18
