# Supplementary material for: Spatial patterns of immunogenetic and neutral variation underscore the conservation value of small, isolated American badger populations
Source: Evol Appl. 2016 Aug 21;9(10):1271–84. doi: 10.1111/eva.12410 (PMC5108218; doi:10.1111/eva.12410)
Supplement: Supplementary file 6 [file EVA-9-1271-s006.pdf]

Table S4. Model comparisons and parameter estimates of six models of codon evolution based on maximum likelihood for MHC sequences in the American badger.

Model	P	Log-likelihood	Parameter estimates	LRT statistic
M0 (one ratio)	1	-701.69	K = 1.67, ω= 2.317	
M3 (discrete)		-623.61	K=1.811, p0= 0.806, p1= 0.175, p2= 0.018	
	5		ω0=0.023, ω1=11.98, ω2=41.454	
**M0 vs. M3**				**156.15*****
M1a (nearly neutral)	2	-663.58	K=0.989, p0=0.779, p1=0.22,ω0= 0, ω1=1	
M2a (positive selection)	4	-625.89	K=1.756, p0=0.753, p1=0.070, p2=0.176	
			ω0=0, ω1=1, ω2=15.189	
**M1a vs. M2a**				**75.38*****
M7 (beta)	2	-663.64	K=0.989, p= 0.005, q= 0.020	
M8 (beta and omega)	4	-625.08	K= 1.767, p0= 0.825, p1= 0.175, p= 0.005	
			q= 0.049, ω= 15.642	
**M7 vs. M8**				**77.13*****

P = number of parameters in the ω distribution; *K* = estimated transition/transversion rate; ω = selection parameter; p_n = proportion of sites that fall into the ω_n site class; p, q = shape parameters of the β function (for models M7 and M8). LRT statistic was computed using $2(Ln_{mod1}-Ln_{mod2})$ where Ln represents the likelihood of the two compared models (mod1 and mod2); P = 0.0001: ***
