# Supplementary material for: Spatial patterns of immunogenetic and neutral variation underscore the conservation value of small, isolated American badger populations
Source: Evol Appl. 2016 Aug 21;9(10):1271–84. doi: 10.1111/eva.12410 (PMC5108218; doi:10.1111/eva.12410)
Supplement: Supplementary file 7 [file EVA-9-1271-s007.pdf]

Table S5. Population pairwise F_{ST} values for 20 microsatellite loci (below the diagonal) and for MHC DRB-2 (above the diagonal) loci across eight populations of American badger at the northern portion of its range. Values in bold indicates statistically significance after 1000 permutations ($p < 0.05$). Population abbreviations as in Table 3

	TO	EK	AB	SK	MB	UP	LP	ON
TO	-	**0.055**	**0.08**	**0.091**	**0.06**	**0.216**	**0.254**	**0.215**
EK	**0.085**	-	0.02	0.025	0.021	**0.154**	**0.141**	**0.116**
AB	**0.077**	**0.044**	-	0.006	-0.001	**0.117**	**0.201**	**0.165**
SK	**0.09**	**0.034**	0.008	-	-0.005	0.052	**0.123**	**0.088**
MB	**0.082**	**0.037**	**0.011**	0.004	-	0.08	**0.156**	**0.124**
UP	**0.117**	**0.084**	**0.051**	**0.06**	**0.054**	-	**0.145**	**0.101**
LP	**0.123**	**0.093**	**0.072**	**0.065**	**0.063**	**0.042**	-	0.023
ON	**0.278**	**0.24**	**0.219**	**0.218**	**0.201**	**0.196**	**0.13**	-
